# Impact of the Chromatin Remodeling Factor CHD1 on Gut Microbiome Composition of *Drosophila melanogaster*

**DOI:** 10.1371/journal.pone.0153476

**Published:** 2016-04-19

**Authors:** Johanna Sebald, Michaela Willi, Ines Schoberleitner, Anne Krogsdam, Dorothea Orth-Höller, Zlatko Trajanoski, Alexandra Lusser

**Affiliations:** 1 Division of Molecular Biology, Biocenter, Medical University of Innsbruck, Innsbruck, Austria; 2 Division of Bioinformatics, Biocenter, Medical University of Innsbruck, Innsbruck, Austria; 3 I-Med GenomeSeq Core, Biocenter, Medical University of Innsbruck, Innsbruck, Austria; 4 Division of Hygiene and Medical Microbiology, Medical University of Innsbruck, Innsbruck, Austria; Oxford Brookes University, UNITED KINGDOM

## Abstract

The composition of the intestinal microbiota of *Drosophila* has been studied in some detail in recent years. Environmental, developmental and host-specific genetic factors influence microbiome composition in the fly. Our previous work has indicated that intestinal bacterial load can be affected by chromatin-targeted regulatory mechanisms. Here we studied a potential role of the conserved chromatin assembly and remodeling factor CHD1 in the shaping of the gut microbiome in *Drosophila melanogaster*. Using high-throughput sequencing of 16S rRNA gene amplicons, we found that *Chd1* deletion mutant flies exhibit significantly reduced microbial diversity compared to rescued control strains. Specifically, although *Acetobacteraceae* dominated the microbiota of both *Chd1* wild-type and mutant guts, *Chd1* mutants were virtually monoassociated with this bacterial family, whereas in control flies other bacterial taxa constituted ~20% of the microbiome. We further show age-linked differences in microbial load and microbiota composition between *Chd1* mutant and control flies. Finally, diet supplementation experiments with *Lactobacillus plantarum* revealed that, in contrast to wild-type flies, *Chd1* mutant flies were unable to maintain higher *L*. *plantarum* titres over time. Collectively, these data provide evidence that loss of the chromatin remodeler CHD1 has a major impact on the gut microbiome of *Drosophila melanogaster*.

## Introduction

The model organism *Drosophila melanogaster* has been used extensively to study the various facets of host-microbe interaction and defence mechanisms. Aside from the discovery of several highly conserved mechanisms regulating the complex networks that underlie the innate immune system of the fly [1], the complex interplay between the host animal and its associated microbiota has been attracting rising attention [1–5]. Results of many studies that analyzed the intestinal microbiota of flies caught in the wild or reared in the laboratory showed that the bacterial community associated with the fly gut is of low diversity, usually ranging from only four to eight species [6–13]. Although, a recent comprehensive analysis of *Drosophila* gut commensals did not find evidence for a “core”microbiome shared across diverse *Drosophila* hosts [13], most of the bacterial species commonly found within the fly gut belong to the genera *Lactobacillus* and *Acetobacter* [10,13]. In light of the relative simplicity of its microbiome compared to the complexity and species richness found in mammals [14], the fly is a highly useful model organism for the study of host-microbe interactions.

In *Drosophila*, just as in mammals, diet is a substantial determinant of intestinal microbiota composition [11,12,15,16], whereas factors like host species identity or phylogeny seem to be of minor importance [12,13]. Wong and colleagues [10] reported that the composition of the gut microbiome changes during *Drosophila* lifespan, probably due to changing physiological conditions in the fly gut. Moreover, the overall bacterial load was shown to undergo fluctuation depending on developmental stage, physiological constitution or immune status of the fly [17–21]. Host-specific genetic factors are further determinants of intestinal microbiome composition. Microbial homeostasis in the gut of *Drosophila* is controlled by the IMD pathway that signals to the NFκB-related transcription factor Relish [1]. Chronic overactivation of the IMD pathway in the gut leads to overexpression of antimicrobial peptide (AMP) genes and has strong deleterious effects. For instance, genetic inactivation or depletion of various negative regulators of IMD signalling and AMP expression causes dysbiosis and ultimately the death of the flies ([2], and references therein). NFκB-mediated gene regulation in vertebrates was shown to involve epigenetic and chromatin remodeling mechanisms to control immune response [22]. Comparably little is known to date about the impact of chromatin-related mechanisms on host defence and gut-microbiome homeostasis in *Drosophila*. However, a recent study demonstrated that AMP expression in response to immune challenge requires the cooperation of Relish with the Brahma-associated protein complex (BAP) via the cofactor Akirin [23]. BAP, which contains the SWI/SNF-related ATPase Brahma (Brm), is a chromatin remodeling factor with a wide and diverse spectrum of functions in *Drosophila* [24].

We have previously shown that another SWI/SNF-type chromatin remodeling factor, CHD1, is involved in *Drosophila* immune response. *Chd1*-mutant flies exhibited increased sensitivity towards oral infection with the gram negative bacterium *Pseudomonas aeruginosa* and showed substantially increased levels of intestinal AMP expression as well as increased bacterial load in the absence of bacterial challenge [25]. Here we investigated in detail the effects of *Chd1* depletion on gut microbiome composition. We find that loss of CHD1 results in a striking reduction of gut microbiome diversity.

## Materials and Methods

### Fly samples

Flies were reared at 25°C and 60% humidity in a 12/12 h light/dark cycle in batches of 25 flies on sugar-cornmeal media (110 g/l refined sugar, 52 g/l cornmeal, 27.5 g/l brewer’s yeast, 4 g/l agar, 2.4 g/l tegosept dissolved in ethanol). The media was boiled for 15 min prior to portioning into sterile plastic vials. Flies were transferred to fresh vials every three days. *Chd1* deficient flies were obtained by crossing flies bearing the deletion allele *Df(2L)Chd1*^*1*^ with the chromosomal deficiency *Df(2L)Exel*^*7014*^, which uncovers *Chd1* [26]. Both alleles are in the *w*^*1118*^ genetic background. In the following, the transheterozygous combination of *Df(2L)Chd1*^*1*^*/Df(2L)Exel*^*7014*^ will be referred to as *Chd1*^*-/-*^. In all experiments the mutant flies were compared to a *Chd1*-mutant strain that was rescued by reintroduction of a wild-type *Chd1* transgene controlled by its native promoter (genotype: *w*^*1118*^*; Df(2L)Chd1*^*1*^,*P{Chd1*^*WT*^*}/ w*^*1118*^*; Df(2L)Exel*^*7014*^,*P{Chd1*^*WT*^*};* in the following referred to as *Chd1*^*WT/WT*^ flies). The origin of these strains is described in detail in [27].

### Amplicon sequencing of 16S rDNA

For gut-specific microbiome analyses, virgin female *Chd1*^*-/-*^ and *Chd1*^*WT/WT*^ flies were collected from different vials of crosses of heterozygous parents as described above. Crosses for both genotypes were handled side-by-side. Virgins were used to be able to compare results with our previous study of infection susceptibility of *Chd1*^*-/-*^ flies [25]. Flies were kept in separate food vials (25 each) until 4 days old and subsequently pooled. Three batches of 10 flies each were randomly collected from the pools and processed for further analysis. To this end, flies were surface sterilized by washing twice with 70% EtOH, and subsequently guts (foregut to hindgut without Malpighian tubules) were dissected in sterile PBS and transferred into 200 μl sterile TES buffer (10 mM Tris-HCl pH 7.6, 1 mM EDTA, 0.5% SDS (w/v)) using sterilized forceps. For a negative control, 200 μl TES buffer without guts was further processed in an identical manner as the gut samples. Isolation of genomic DNA was performed as described [25]. DNA was dissolved in 25 μl sterile a.d. and 1 μl (approximately 200 ng DNA) was used as template for 16S rDNA amplification. Bacterial universal primers *27F* (5’-AGAGTTTGATCMTGGCTCAG-3’) and *R361* (5’-CTGCTGCCTCCCGTAGGAG-3’) [modified from lifetechnologies.com/microbial] covering the variable regions V1 and V2 of the bacterial 16S rRNA gene were used for library preparation. Adapter sequences, required for bidirectional sequencing, as well as sample-specific barcodes were fused to the target-specific primers according to the manufacturers’ instructions (Ion Amplicon Library Preparation User Guide; Life Technologies). The 16S amplicon libraries were purified using Agencourt AMPure XP reagent (Beckman Coulter). Template preparation was performed using the Ion One Touch^TM^ 2 System and the Ion PGM^TM^ Template OT2 400 Kit (Life Technologies). Sequencing was performed using the Ion PGM^TM^ Sequencing 400 Kit on the Ion PGM^TM^ System (Life Technologies). Barcoded samples were first run on a 314^TM^ Chip v2 (Life Technologies) and afterwards on a Ion 316^TM^ Chip v2 (Life Technologies). Sequences of both Chips were pooled for the respective sample replicates.

### Bioinformatics analyses

For bioinformatic analysis of deep sequencing samples and data visualization the open-source software QIIME (Quantitative Insights Into Microbial Ecology; version 1.8.0; [28] and R (version 3.1.1; [29]) were used. Demultiplexing and quality filtering was done according to the following requirements: minimum quality score of 20 in each 50 bp sliding window, minimum length of 150 bp, homopolymers no longer than 6 bp, as well as a maximum number of six ambiguous bases. Preprocessing of the sequences resulted in a mean sequence length of 272.57 +/- 32.33 bp. For subsequent clustering of the preprocessed sequences into operational taxonomic units (OTUs) the open reference approach was selected. OTU picking was carried out with an identity of 97% using the usearch61 algorithm [30] with the SILVA database (version 111; [31]) as reference. This script includes further steps, such as taxonomy assignment with UCLUST ([30]; 90% similarity; SILVA reference database and default settings), sequence alignment using the PyNAST algorithm (default settings; [32]), phylogenetic construction applying FastTree [33]. Quality filtering of the OTUs was performed according to the following requirements: removal of OTUs with a sequence count lower than 0.005% of total sequence number and total observation count <2. OTU assignments for the individual replicates before and after filtering are shown in S1 Table. To create S2 Table, results from individual replicates were merged using the Python script *summarize_otu_by_cat*.*py* provided by QIIME. Where indicated in the main text and figures, alternative filtering parameters were used: (i) Removal of OTUs with a sequence count <10 and of OTUs with higher number of reads in the negative control than in the sample [10]. (ii) Removal of OTUs with a sequence count lower than 0.005% of total sequence number and total observation count <2 and of OTUs with higher number of reads in the negative control than in the sample. Alpha- and jackknifed β-diversity (normalized, weighted UniFrac distances) were calculated in QIIME and the corresponding plots illustrated using R and the *phyloseq* package [34]. For statistical analysis of the α-diversity indices, Welch two-sample t-test was applied in R. “Effective number of species” was calculated from Shannon index (SI) values (e^SI^). Statistical significance of the UniFrac distances was analysed by a two sample t-test within QIIME. The heatmap, showing the 25 most abundant 97% identity OTUs, was created in R using the *gplots* package [35]. Relative abundance was calculated based on the sequence counts per OTU, values were transformed into log10 scale. Hierarchical clustering using a euclidean distance measure was performed in R. The alignment against all *Acetobacter* (1294) and *Lactobacillus* (46782) reference sequences deposited in the SILVA database was performed in QIIME using the PyNAST algorithm [32]. The confidence level was set at 99%, sequences with fewer than 10 counts were excluded from the analysis. Sequencing data is available at the Short Reads Archive (SRA) accession number SRP064451.

### Bacterial plating assays

For bacterial plating assays, virgin female *Chd1*^*-/-*^ and *Chd1*^*WT/WT*^ flies were reared on standard food (see above) with transfer to fresh food vials on every third day. At the respective ages of 4, 7, 10, 14, 21 days, flies were collected and consecutively washed with 10% bleach, 70% ethanol, and three times with sterile 1xPBS (Dulbecco). Since *Chd1*^*-/-*^ flies show a three-day delay in eclosion timing compared to *Chd1*^*WT/WT*^ flies (S1 Fig), crosses were set up such that mutant and control flies would eclose at the same time and could be aged in parallel. Batches of 6 surface-sterilized flies were homogenized in 100 μl sterile 1xPBS using autoclaved microtube pestles. Homogenates were cultured on Ace agar (0.8% (w/v) yeast extract, 1.5% (w/v) peptone, 1% (w/v) dextrose, 0.3% (v/v) acetic acid, 0.5% (v/v) ethanol; [21,36]) or MRS agar (2 g/l di-ammonium hydrogen citrate, 2 g/l Na_2_HPO_4_, 20 g/l D(+)-glucose, 0.1 g/l MgSO_4_, 0.05 g/l MnSO_4_, 0.05 g/L, 5 g/l meat extract, 5 g/l sodium acetate, 10 g/l universal peptone, 5 g/l yeast extract, 12 g/l agar) at 27.5°C (Ace) or 37°C (MRS) for 3 to 4 days. MRS plates were incubated under microaerophil conditions. Two technical replicates were plated of serial dilutions of four biological (6 flies each) replicates at each time point. CFUs were counted and mean numbers of bacteria per fly were calculated.

### Conventional and real-time PCR analyses

Gut preparation from virgin females at different ages (4, 7, 10, 14 days old) and DNA extraction were performed as described above for amplicon sequencing. DNA was isolated from pools of ten guts each. For detection of *Acetobacteraceae* by end-point PCR, 17 ng DNA was used with primers Ace1 (5’-CZAGTGTAGAGGTGAAATT) and Ace2 (5’-CCCCGTCAATTCCTTTGAGTT) at an annealing temperature of 55°C; for detection of *Pseudomonadaceae* by end-point PCR 200 ng DNA and primers Pse1 (5’-TCCAAGTTTTAAGGTGGTAGGCTG) and Pse2 (5’-CTTTTCTTGGAAGCATGGCATC) at an annealing temperature of 60°C was used. Three PCR reactions (25, 30, 35 cycles for detection of *Acetobacteraceae* and 30, 35, 40 cycles for *Pseudomonadaceae*) of each of three biological replicates were performed for each time point. PCR products were separated on 2% agarose gels and intensities of ethidium bromide-stained bands were quantified using Fiji for MacOS X. The ratio of the intensities of *Pseudomonas* and *Acetobacter* bands of the same DNA sample was determined. Results are shown as mean ratios ± SEM. For qPCR analysis of *L*. *plantarum*, 100 ng DNA was used with the *L*. *plantarum*-specific primers Lp16Sf (5’-TGATCCTGGCTCAGGACGAA-3’) and Lp16Sr (5’-TGCAAGCACCAATCAATACCA-3’) [37]. qPCR reactions were conducted in triplicate using a StepONEPlus instrument (Life Technologies) and Power SYBR Green PCR master mix (Life Technologies). The Drosophila GAPDH gene was used for normalization and 2^−ΔCT^ values were plotted.

### Dietary supplementation with *L*. *plantarum*

Whatman filter discs were soaked in 2 ml 5% sucrose containing 10^7^ cfu of *L*. *plantarum*. The discs were placed into 200 ml vials and batches of 20 four-day old virgin female flies that had been starved for 24 h in vials containing filter discs soaked with 1x PBS were added to each vial (3 replicates per line). The flies were kept overnight in the inoculated vials at 25°C and subsequently transferred into fresh food vials every 3^rd^ day. *L*. *plantarum* load was analysed at days 1, 4, 7, 10, 14 and 21 post inoculation by qPCR and by plating of fly-homogenate on MRS agar as described above. Statistical significance was determined using Student’s t-test (Prism 5.0).

## Results

### Loss of CHD1 leads to a significant reduction of bacterial diversity in the gut of the flies

**Fig 1:**
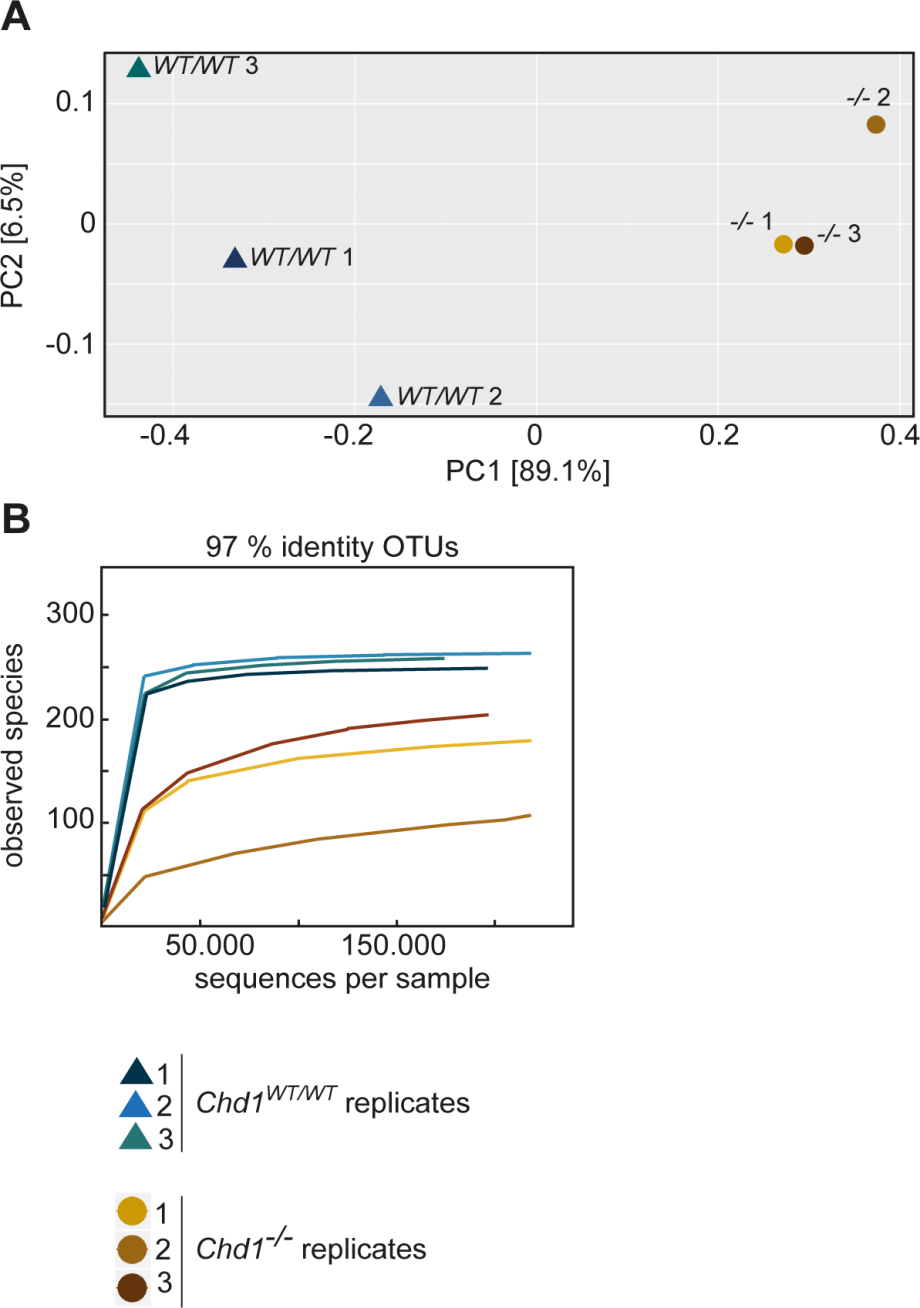
The microbiome composition in the gut of *Chd1*^*-/-*^ and *Chd1*^*WT/WT*^ flies is significantly different. (A) Principal Coordinate Analysis (PCoA) plot depicting β-diversity by jackknifed UniFrac distances (normalized, weighted UniFrac metric) based on 97% similarity OTU assignments. *Chd1*^*WT/WT*^ and *Chd1*^*-/-*^ replicates differ considerably for PC1, which explains 89.1% of the total variation. To estimate the statistical significance of the clustering a two-sample t-test based on distance matrices (distances within all replicates versus distances between all replicates) was performed (P = 4.709 e-5). (B) Rarefaction curves of 97% identity OTUs for *Chd1*^*WT/WT*^ and *Chd1*^*-/-*^ sample replicates show exhaustive sampling depth.

To examine the composition of the intestinal microbiome of *Chd1* null flies, we performed deep sequencing of 16S rRNA gene amplicons of guts isolated from *Chd1*^*-/-*^ and control flies (*Chd1*^*WT/WT*^), which carried a wild-type *Chd1* rescue transgene in a *Chd1*-deficient genetic background [27]. We isolated genomic DNA from guts of 4 days old virgins for PCR amplification of the variable regions V1 and V2 of the bacterial 16S rRNA gene. Using the Ion PGM sequencing platform for deep sequencing, we obtained a total of 1,451,602 reads after quality filtering representing a mean 241,933 reads per replicate. The preprocessed sequences were clustered into operational taxonomic units (OTUs) using the SILVA database [31] as a reference with a sequence identity threshold of 97%. Principal Coordinate Analysis (PCoA) revealed clear separation between *Chd1* mutant and wild-type replicates with PC1 explaining 89.1% of the overall variation (Fig 1A). This was confirmed by a two-sample t-test applied on the calculated distance matrix using QIIME (P = 4.709 e^-5^). Thus, the difference in bacterial community composition of *Chd1* mutant and wild-type samples correlates with the genetic background of the host flies, whereas variability among replicates of the same genotype only accounts for a minimal portion of the total disparity.

The rarefaction curves for the OTUs assigned to *Chd1* mutant and *Chd1*^*WT/WT*^ gut samples reached saturation for the *Chd1*^*WT/WT*^ samples, and near-complete saturation (power estimate above 99%) for the mutant, indicating that only few, very rare OTUs might remain undetected in the mutant gut samples at this sequencing depth (Fig 1B). The combined reads of wild-type and mutant samples were assigned to a total of 273 OTUs. While all OTUs were found in the combined wild-type samples, only 238 (87%) were present in *Chd1*^*-/-*^ flies (S2 Table). Furthermore, estimated species richness was about 1.3 times higher in *Chd1*^*WT/WT*^ compared to *Chd1*^*-/-*^ samples (Chao1 richness estimate, Table 1). Shannon diversity index calculation revealed a significantly lower value for *Chd1*^*-/-*^ guts compared to *Chd1*^*WT/WT*^ guts (Table 1), which translates into at least 7 times lower effective species diversity in the mutant.

**Table 1:** Chao1 and Shannon indices of *Chd1* mutant and wild-type sample replicates. Three sets of filtering parameters were used to analyse the data.

		*filtering parameters 1*^*1*^			*filtering param*. *2*^*2*^		*filtering param*. *3*^*3*^	
		*mean number of sequences*	*Chao1 species richness estimate*	*Shannon diversity index*	*Chao1*	*Shannon*	*Chao1*	*Shannon*
	R1	217,999	250.43	3.37	927.71	3.75	227.43	3.38
	R2	200,030	269.5	2.91	939.22	3.27	274.0	2.92
***Chd1***^***WT/WT***^	R3	243,807	267.33	4.33	828.6	4.63	247.20	4.34
	*mean*	**220,612**	**262.42**	**3.54**	**898.51**	**3.88**	**249.54**	**3.55**
	R1	232,649	206.25	2.04	636.64	2.12	196.25	2.01
	R2	342,146	195.07	1.3	367.67	1.34	173.13	1.30
***Chd1***^***-/-***^	R3	214,971	214.12	1.45	664.44	1.44	197.55	1.38
	*mean*	**263,255**	**205.15**	**1.60**	**556.25**	**1.63**	**188.97**	**1.57**
***P-value***	*<0*.*05*	** **	0.00225**	0.02535*	**0.05475**	0.01312*	0.02667*	0.02379*

^1^ discard OTUs with <0.005% of total # of reads and OTUs with reads in <2 replicates.

^2^ discard OTUs with <10 reads and OTUs with higher # of reads in blank compared to sample [10].

^3^ discard OTUs with <0.005% of total # of reads and OTUs with reads in <2 replicates and OTUs with higher # of reads in blank.

* P-value < 0.05

** P-value < 0.01

Since *Chd1*^*-/-*^ flies have elevated bacterial titres in the gut [25], it was formally possible that the diversity difference between the two strains was caused by preferred detection of contaminating species in the wild-type sample due to the lower bacterial load in the starting material. Sequencing of the negative control sample revealed the presence of similar OTUs as in the actual samples. However, the amplicon yield from “blank” samples was very low compared to the actual samples and therefore is unlikely to contribute significant numbers of reads in the gut samples (see S2 Fig for an amount-specific comparison of species distribution). Note that the number of reads obtained from the “blank” or the actual samples (S2 Table) does not correspond to absolute bacterial amounts, since quantitative differences in the input DNA are equalized through library preparation. For a quantitative comparison between sample groups the actual differences in total amount of bacteria retrieved from each gut-sample has to be taken into account, which is visualized in S2 Fig Moreover, the “blank” sample contained fewer OTUs (234) than the wild-type (273) and the mutant samples (238).

Different procedures for generating and processing data of high-throughput microbiome analyses exist to date. Moreover, the fly microbiome is affected by various biological and environmental effects, such as diet, sex, age, developmental state and possibly others (see introduction). With these caveats in mind, we note that the average Chao’s richness estimate and the average Shannon index of diversity for *Chd1*^*WT/WT*^ and *Chd1*^*-/-*^ samples were higher than previously reported for lab-reared flies (e.g. [10,12]). To assess, if our data analysis parameters might contribute to the increased alpha-diversity in our samples, we re-analysed the data using the filtering parameters published by Wong *et al*. [10]. Thereby, all OTUs with <10 reads and all OTUs for which read number was higher in the negative control than in the sample were considered contaminants and discarded. The less stringent parameters (our original analysis removed sequences with a count lower than 0.005% of total sequence number and total observation count <2) increased the number of detected OTUs. Taxa distribution and diversity indices, however, remained very similar for *Chd1* wild-type and mutant samples (Table 1, S3 Fig). Likewise, filtering of OTUs with <0.005% reads, singletons and OTUs with higher read numbers in the negative control did not significantly change the Shannon index (Table 1).

We also performed downsampling of our datasets to 100 or 5000 reads/sample to account for potential differences due to sampling depth. Rarefaction curves indicated that datasets with a 5000 reads/sample cap are sufficiently representative, and species richness and species diversity indices were quite similar to those of full datasets. By contrast, rarefaction curves of datasets capped to 100 reads/sample did not reach saturation and the differences of diversity index values were no longer statistically significant between *Chd1*^*WT/WT*^ and *Chd1*^*-/-*^ guts (S4 Fig, S2 Table).

In summary, the results provide evidence for an influence of CHD1 on the commensal community composition in the gut of *Drosophila* in that loss of this chromatin remodeler results in a reduction of species diversity of intestine-associated bacteria.

### *Acetobacter* sp. account for more than 98% of intestinal bacteria in *Chd1*^*-/-*^ flies

**Fig 2:**
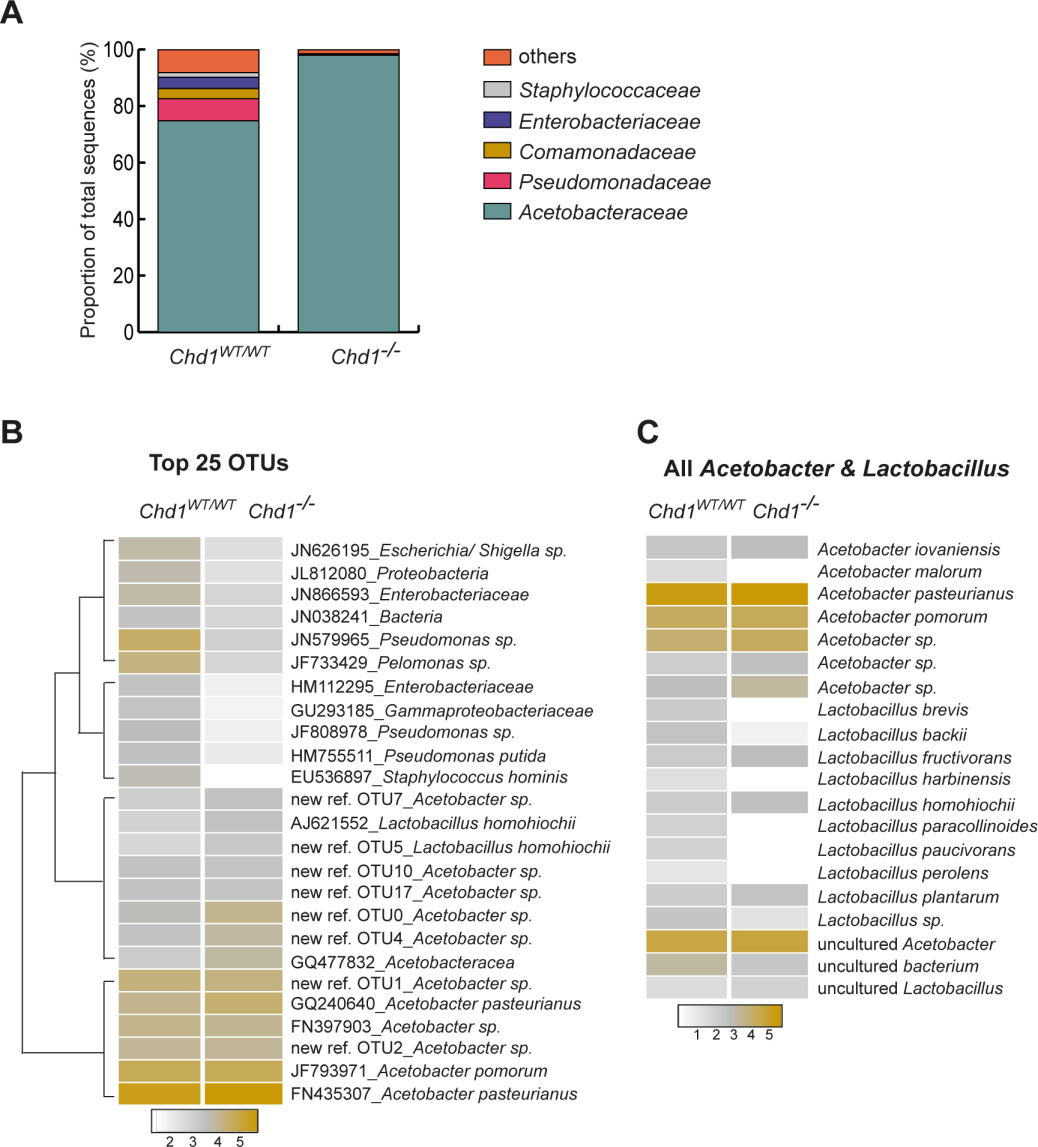
Loss of CHD1 causes decreased species diversity in the gut microbiome. (A) Relative abundance of bacterial families (97% similarity threshold) determined by 16S rDNA sequencing in *Chd1*^*WT/WT*^ and *Chd1*^*-/-*^ samples. Families present at levels less than 1.5% were summarized as “others“. (B) Heatmap of the 25 most abundant 97% identity OTUs within *Chd1*^*WT/WT*^ and *Chd1*^*-/-*^ guts. OTU classification down to the lowest level possible is shown. Color bars denote the relative abundance (log10 values) of each OTU within the respective sample. OTUs are clustered according to their average relative abundance. (C) Heatmap showing the abundance of *Acetobacter* and *Lactobacillus* species in *Chd1*^*WT/WT*^ and *Chd1*^*-/-*^ samples identified by alignment of sequencing reads to all respective sequences in the SILVA database at an identity threshold of 99%. All OTUs with fewer than 10 counts, were excluded. Color bars denote the relative abundance (log10 values) of each species within the respective sample.

Investigation of the bacterial taxa associated with the gut of *Chd1* mutant and wild-type flies revealed that at the phylum level, *Proteobacteria* accounted for more than 97% of total reads across all samples, whereas *Firmicutes*, which have been shown to normally be the second dominating phylum in adult flies [10,13], were strikingly underrepresented in our analysis. On the family level, the bacterial community associated with *Chd1*^*WT/WT*^ guts was dominated by *Acetobacteraceae*, while *Pseudomonadaceae*, *Enterobacteriaceae*, *Comamonadaceae* and *Staphylococcaceae* together comprised ~19% of the microbiota. By contrast, these families were nearly absent (0.5%) in *Chd1*-mutant flies. Likewise, *Lactobacillaceae*, accounted for only 0.3% within the bacterial community of wild-type and 0.2% of that of mutant flies (Fig 2A, S3 Fig, Table 2). All of these families have been reported to be associated with *Drosophila* before [11]. The relative proportion of detected bacterial families changed only slightly when the above-described different filtering parameters were applied (S3 Fig, Table 2). We next performed cluster analysis of the 25 most abundant OTUs (classification down to the lowest level possible) across both samples. Consistent with the taxa summary (Fig 2A), OTUs assigned to the *Pseudomonadaceae*, *Enterobacteriaceae*, *Comamonadaceae* or *Staphylococcaceae* families grouped into the two clusters with the lowest relative abundance, whereas *Acetobacteraceae* dominated the two high abundance OTU clusters (Fig 2B). The differences between *Chd1* mutant and wild-type samples were most pronounced in the two low abundance clusters, whereas the variations in the *Acetobacter*-dominated clusters were minor.

**Table 2:** Distribution of bacterial families within the gut microbiome of *Chd1* mutant and wild-type flies. Three sets of filtering parameters were used to analyse the data.

	filtering param. 1^1^		filtering param. 2^2^		filtering param. 3^3^	
	*Chd1*^*WT/WT*^	*Chd1*^*-/-*^	*Chd1*^*WT/WT*^	*Chd1*^*-/-*^	*Chd1*^*WT/WT*^	*Chd1*^*-/-*^
*Acetobacteraceae*	73.9%	98.1%	72.1%	98.6%	73.4%	98.8%
*Pseudomonadaceae*	7.7%	0.2%	7.9%	0.3%	7.9%	0.2%
*Comamonadaceae*	4.4%	0.2%	4.6%	0.2%	4.6%	0.2%
*Enterobacteriaceae*	4.4%	0.3%	4.4%	0.3%	4.4%	0.3%
*Staphylococcacea*	1.6%	0.0%	1.6%	0.0%	1.6%	0.0%
*Moraxellaceae*	1.0%	0.1%	1.2%	0.1%	1.0%	0.1%
*Methylobacteriaceae*	0.6%	0.0%	0.7%	0.0%	0.6%	0.0%
*Caulobacteraceae*	0.6%	0.0%	0.7%	0.0%	0.6%	0.0%
*Propionibacteriaceae*	0.3%	0.0%	0.3%	0.0%	0.3%	0.0%
*Lactobacillaceae*	0.3%	0.2%	0.3%	0.0%	0.2%	0.0%
others	5.2%	0.9%	6.1%	0.3%	5.5%	0.3%

^1^ discard OTUs with <0.005% of total # of reads and OTUs with reads in <2 replicates. Observed species: 246 (wt)/159 (mut).

^2^ discard OTUs with <10 reads and OTUs with higher # of reads in blank compared to sample [10]. Observed species: 831 (wt)/415 (mut).

^3^ discard OTUs with <0.005% of total # of reads and OTUs with reads in <2 replicates and OTUs with higher # of reads in blank. Observed species: 237 (wt)/148 (mut).

Although there appears to be no “core”microbiome in *Drosophila* [10], there are five bacterial species that are often prevailing in the intestinal microbiota: *Acetobacter pomorum*, *A*. *tropicalis*, *Lactobacillus brevis*, *L*. *plantarum* and *L*. *fructivorans* [8–10,13,17,38,39]. Of these five taxa we only found *A*. *pomorum* to be present among the 25 most abundant OTUs within *Chd1* mutant and wild-type guts (Fig 2B). *A*. *pomorum* and *A*. *pasteurianus*, a constituent of the fly surface as well as intestinal microbiota [8,17], were the two dominating species within both, *Chd1*^*WT/WT*^ as well as *Chd1*^*-/-*^ guts (Fig 2B). These results are in agreement with the finding that *A*. *pomorum/pasteurianus* have been found in most laboratory *Drosophila* stocks but not being laboratory-specific like several *Enterococcaceae* [8,39] and *Enterobacteriaceae* [11]. Moreover, we detected *Staphyloccocus hominis*, a constituent of *Drosophila* surface bacteria, the saprophytic soil bacterium *Pseudomonas putida*, a close relative of the fly pathogen *P*. *entomophila* [40,41] and *L*. *homohiochii*, a reported member of the fly microbiome [17] among the top 25 OTUs (Fig 2B).

Given that *Acetobacter* and *Lactobacillus* species are frequent commensals in the gut of *Drosophila*, we examined in more detail all *Acetobacter* and *Lactobacilli* identified in the microbiota of *Chd1* mutant and wild-type flies by alignment to sequences deposited in the SILVA database using a 99% identity threshold. All *Acetobacter* species present in our samples (Fig 2C) have been previously reported to associate with *Drosophila*. By contrast, with the exception of *L*. *brevis*, *L*. *plantarum*, *L*. *fructivorans* and *L*. *homohiochii*, none of the other identified *Lactobacillus* species has been linked to the *Drosophila* microbiome before. Comparison of *Chd1*^*WT/WT*^ and *Chd1*^*-/-*^ guts revealed that while there was no major variation in the relative abundance of the identified *Acetobacter* species, several *Lactobacilli* were present at significantly lower levels or even absent in *Chd1* mutants. Only *L*. *fructivorans*, *L*. *homohiochii* and *L*. *plantarum* showed similar levels as in the wild type (Fig 2C).

### Bacterial titres increase more quickly with age in the guts of *Chd1*^*-/-*^ flies

**Fig 3:**
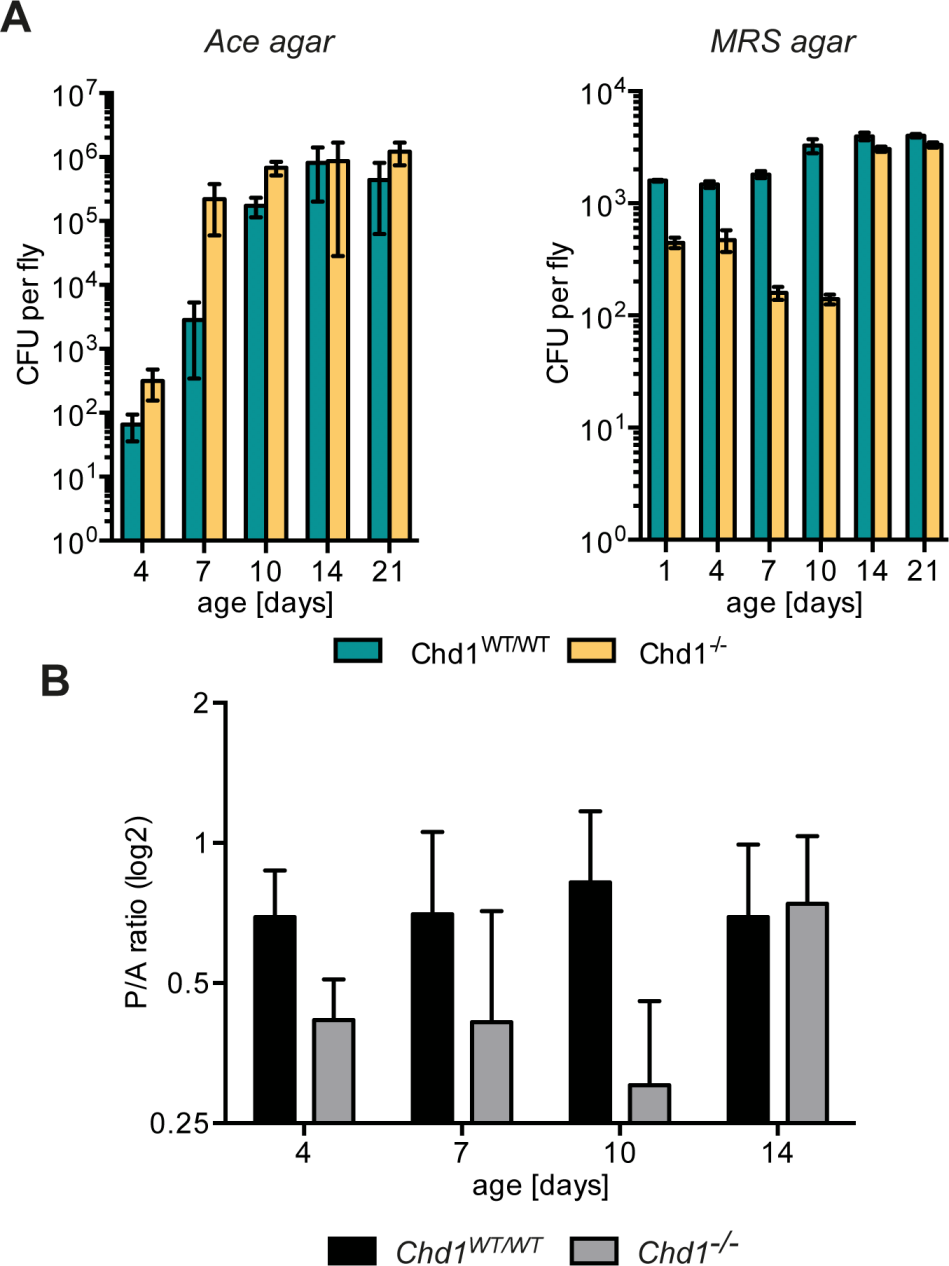
Age-related changes of bacterial load and species distribution in *Chd1* mutant and wild-type flies. (A) Flies of the indicated ages were surface-sterilized and homogenates were plated on Ace agar (left panel) to select for *Acetobacteraceae* or on MRS agar (right panel) to select for *Lactobacillaceae*. CFUs per fly were calculated and mean values from four biological replicates ±SEM were plotted. (B) Semiquantitative PCR was used to determine the relative amounts of *Acetobacter* “A” and *Pseudomonas* “P” species in guts of flies of the indicated ages. Band intensities were quantified and P/A ratios were calculated. Mean values ±SEM of three biological replicates are shown.

To determine if differences in gut microbiome composition and titre between *Chd1*^*-/-*^ and *Chd1*^*WT/WT*^ flies persist during adult life, we employed PCR- and bacterial plating assays. Since our deep sequencing results showed that the majority of bacteria in both *Chd1* mutant and control strains belongs to the *Acetobacteraceae* family, we plated *Drosophila* homogenates on the Ace medium semiselective for *Acetobacteraceae* [21] for estimation of bacterial numbers. Moreover, we used plating on MRS agar under microaerophil conditions to assay *Lactobacillus* load. In good agreement with earlier reports [17–19,21,42], bacterial titres increased with age in *Chd1*^*WT/WT*^ flies but reached a plateau around 14 days of age. *Chd1* mutant guts exhibited considerably higher *Acetobacter* titres at the earlier time points tested (4, 7, 10 days), while titres were roughly equal in mutant and control flies at days 14 and 21 (Fig 3A, left panel). Particularly on day 7, bacterial load was almost two orders of magnitude higher in *Chd1*^*-/-*^ flies compared to *Chd1*^*WT/WT*^ suggesting rapid accumulation of *Acetobacteraceae* in younger mutant flies. By contrast, *Lactobacilli* showed lower representation in younger *Chd1* mutant flies compared to rescued control flies with progressively decreasing titres until day 10 of age. At days 14 and 21, *Chd1* mutant and wild-type flies had again similar *Lactobacillus* levels (Fig 3A, right panel). These results confirm our results from the deep sequencing experiments (performed with 4 day old flies) that the loss of *Chd1* correlates with an accumulation of *Acetobacter* and a decrease of *Lactobacillus* species in *Drosophila*.

### Increased dominance of *Acetobacteraceae* in guts of *Chd1*^*-/-*^ flies at different ages

Previously it was shown that *Drosophila* gut microbiome composition is variable and can differ considerably even between individual flies of the same strain [13]. To further rule out that the species distribution differences we detected by deep-sequencing in *Chd1* mutant and wild-type flies were a result of sampling all biological replicates from one fly generation, and to further corroborate the changes in relative distribution of *Acetobacteraceae* and other bacterial families (as also observed in Fig 3A), we sought to determine the relative amounts of *Acetobacteraceae* (the major family in both strains) and *Pseudomonadaceae* (~8% in *Chd1*^*WT/WT*^ vs 0.2% in *Chd1*^*-/-*^) in further samplings of the two fly strains. To this end, we collected guts from a new generation of both strains (about two years after the original sampling for the deep-sequencing analysis). Moreover, we collected guts from flies of different ages (4, 7, 10, 14 days old) and analysed the samples using semiquantitative PCR with taxa-specific primers. We had to resort to this method, as we were unable to find *Pseudomonadaceae*-specific primers that were suitable for real-time PCR. Within the limitations of this methods regarding accuracy, we found that in most *Chd1*^*-/-*^ samples the fraction of *Pseudomonas* species was lower than in the control fly guts (Fig 3B). As observed with the plating assays for *Acetobacter* and *Lactobacillus* (Fig 3A), the differences between mutant and control guts disappeared also for the *Acetobacter/Pseudomonas* ratio at 14 days of age.

Collectively, the results show that loss of CHD1 does not affect the core species present under our cultivation conditions but rather contributes to the relative reduction or elimination of minor bacterial taxa. The data also suggest that this effect shows its strongest manifestation in young flies, while it seems that upon aging the microbiomes of mutant and control flies become more similar to each other.

### *Chd1*^*-/-*^ flies are unable to sustain *Lactobacillus plantarum* titres after dietary supplementation

**Fig 4:**
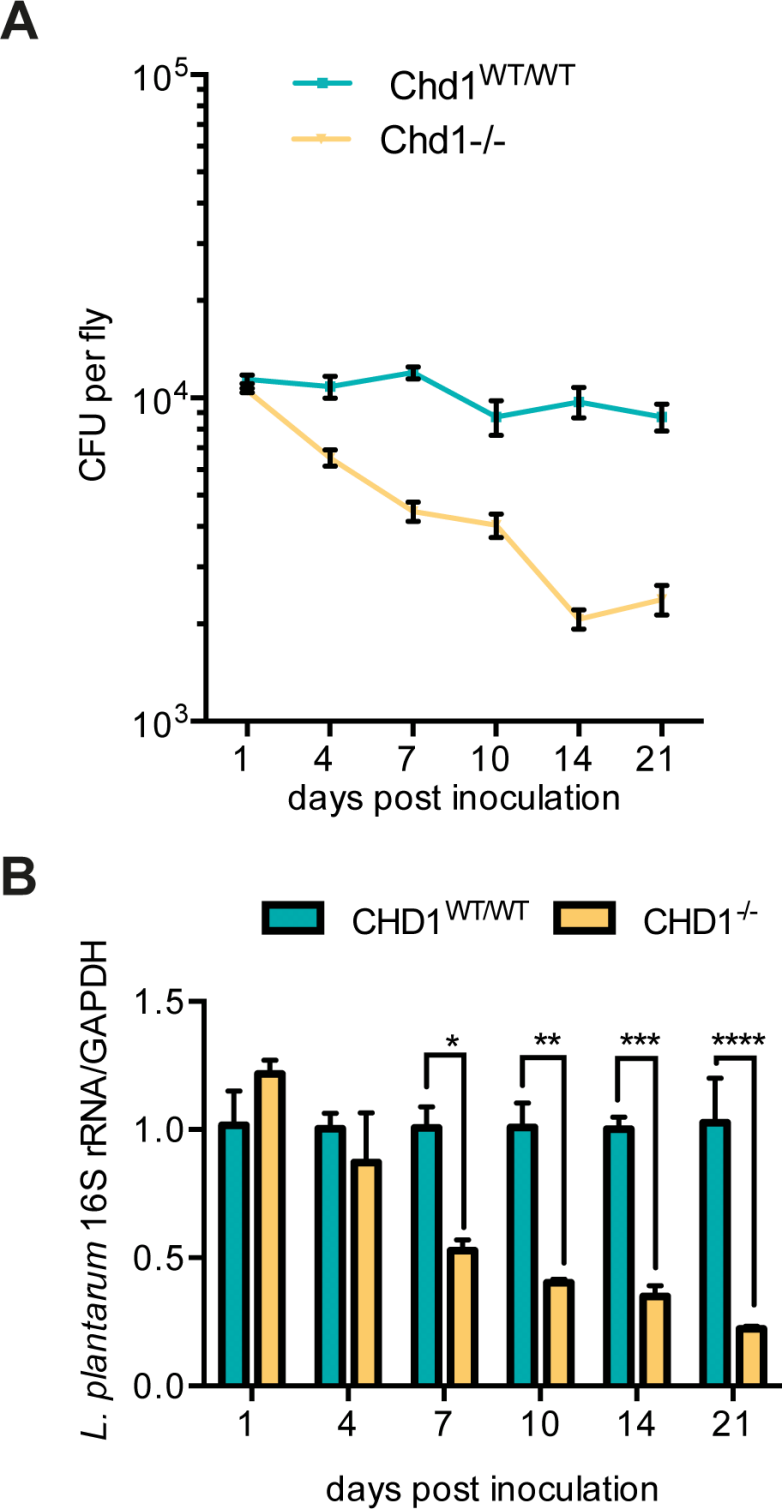
Time-dependent decrease in supplemented *L*. *plantarum* in *Chd1*^*-/-*^ but not *Chd1*^*WT/WT*^ flies. Flies were fed on *L*. *plantarum* overnight, transferred to fresh food vials and collected at the indicated days after inoculation. (A) *Lactobacillus* load was determined by plating fly homogenates on MRS agar; (B) *L*. *plantarum* was detected by real-time PCR. Signals were normalized against the *Drosophila* GAPDH gene and mean values ±SEM of three biological replicates are shown. Significant differences between the two fly lines were analyzed by t-test (level of significance set to p<0.05) and are marked by (*).

Our plating assays showed that *L*. *plantarum* levels were already decreased in one-day old *Chd1* mutant flies compared to wild-type flies. To determine, if elevating *Lactobacillus* levels in young flies would prevent its observed diminishment in the guts of *Chd1*^*-/-*^ flies at later stages of life, we fed 4-day old *Chd1* mutant and control flies with a bacterial suspension of *L*. *plantarum* overnight, subsequently transferred the flies to standard food vials, and determined *Lactobacillus* titres by plating fly homogenates on MRS agar and by qPCR with *L*. *plantarum*-specific primers. Both assays clearly showed that while *L*. *plantarum* load did not significantly change over a period of 21 days in control flies, it decreased continuously in *Chd1* mutant flies despite equal initial titres in both strains (Fig 4A and 4B). These results suggest that the environment in *Chd1*^*-/-*^ guts discriminates against the growth of bacteria such as *Lactobacillus*, while it promotes accumulation of *Acetobacter* sp.

## Discussion

The *Drosophila* gut microbiome is predominantly shaped by the nutritional environment. Several studies showed that in *Drosophila*, just as in mammals, diet is a substantial determinant of intestinal microbiota composition, whereas factors such as host species identity or phylogeny seem to be of minor importance [11–13,15,43]. The profound contribution of commensal microbiota to mammalian health, in particular with regard to inflammatory diseases of the intestine, is well recognized [44]. We have previously shown that loss of *Chd1* in flies renders them sensitive towards oral infection with *P*. *aeruginosa*. In particular, we found that large numbers of bacteria were able to transit into the body cavity in the course of infection in *Chd1* mutant but not control flies suggesting compromised barrier function of intestinal epithelia [25]. Here we confirm elevated bacterial titre, in particular *Acetobacter* levels, in the gut of *Chd1* mutant flies, and we show that the microbiome of mutant guts is significantly less diverse than that from wild type. Whether this change in bacterial community composition contributes to increased infection sensitivity is not known at this point.

The *Drosophila* gut microbiome, especially that of lab-reared flies, is known to be of low complexity with *Lactobacillus* and *Acetobacter* being the major species [3]. In agreement with earlier reports, our data demonstrate the dominance of *Acetobacter* species in both wild-type and mutant lines. However, *Lactobacilli* represented less than 1% of all identified OTUs. Therefore, the microbiome composition under our rearing conditions resembles that of UCSD stock center flies or flies caught in the wild as shown by Staubach *et al*. [12]. In the reviewing process of this study, it has been suggested that the comparably high alpha diversity obtained with our analysis may in part be due to higher error rates of the Ion Torrent PGM platform that was used for the amplicon sequencing analysis. In a recent comparison of Ion Torrent PGM and the Illumina MiSeq platform, the error rates differed between ~1.5/100 bases for Ion Torrent and 0.9%/100 bases for Illumina sequencing. As these differences are quite small, when analysing microbial community composition, the authors found good concordance of results generated by the two platforms [45]. In fact, MiSeq analysis of the V1-V2 amplicon of 16S rDNA recently was found to give inferior results in a comparison with Ion Torrent amplicon sequencing and shotgun sequencing using different platforms [46]. We cannot rule out that some of the OTUs in our study are false positives. However, our results have gained support by recent work by Clark *et al*. [42], in which a shotgun Illumina sequencing approach uncovered considerable diversity in the *Drosophila* gut microbiome.

With respect to beta diversity, our sequencing data clearly show a shift in microbial community composition between *Chd1* wild-type and mutant fly guts with strongly increased presence of *Acetobacteraceae* relative to other bacterial families leading to changes in alpha and beta diversity measures. At the same time, we observed an increase in bacterial load in *Chd1*^*-/-*^ flies. This increase, which is apparently caused by an overgrowth of *Acetobacteraceae* in young flies (see Fig 3A), in theory might cause a bias against low abundance taxa in the deep sequencing analysis. However, results of the culture-dependent analyses show that *Lactobacillus* titres are lower in the mutant than in the wild-type and further decrease until the age of 10 days. Similar results were obtained for *Pseudomonas* by a PCR-based approach. Finally, there is a striking difference between *Chd1* mutant and wild type flies in their ability to maintain levels of supplemented *L*. *plantarum* suggesting an unfavourable environment for this (and possibly other) bacterial species in the mutant guts. Together these observations argue in favour of decreased diversity in the gut microbiome of *Chd1*^*-/-*^ compared to *Chd1*^*WT/WT*^ flies rather than biased sequencing due to increased *Acetobacter* load.

A handful of host factors has so far been described in *Drosophila* to affect intestinal microbiome composition. For instance, the transcription factor Caudal (Cad) was shown to be necessary for regulating basal expression levels of AMPs, and depletion of Cad resulted in AMP overexpression and dysbiosis [9]. Likewise, the age-dependent increase of dFOXO expression was found to lead to gradual disturbance of intestinal bacterial load by repressing the transcription of the negative IMD-pathway regulator PGRP-SC2 [19].

Little is known, however, about the influence of epigenetic processes on gut and microbiome homeostasis in *Drosophila* and mammals, respectively. Our results provide clear evidence that the chromatin remodeling factor CHD1 affects intestinal microbiome composition in the fly. This may occur through direct regulation of AMP expression in the gut [25], similar to what has recently been shown for the chromatin remodeling complex BAP in the course of infection [23]. For example, it is possible that the higher steady-state AMP levels present in the absence of CHD1 may result in the elimination of potentially more sensitive minor taxa thereby causing the observed overgrowth in terms of diversity as well as bacterial titre of *Acetobacter* spp.

Alternatively, CHD1 might act indirectly, for instance by causing deregulation of gut physiology or developmental processes, which in turn may favour microbiome changes and/or affect susceptibility to infection. Regardless of the way by which loss of CHD1 affects *Acetobacter* titre initially, the further increase with progressing age may be linked to replenishment of the gut microbiome by feeding of the flies on their feces [21]. This may also enforce the dominance of *Acetobacter* over all other species and aggravate the reduction of minor taxa as indicated by the age-linked reduction of *Pseudomonas* compared to *Acetobacter* (Fig 3B). However, why bacterial titres as well as the relative levels of some minor taxa become more similar between mutant and wild-type flies at later stages of age is not known to date. Although there are many possible explanations, it is tempting to speculate that the gut environment of younger *Chd1*^*-/-*^ flies may somehow resemble that of older wild-type flies [17,21,42]. Not only the elevated bacterial load in young *Chd1*^*-/-*^ flies, but also the previously demonstrated higher levels of AMPs provide evidence for this notion, since upregulation of AMP genes was detected in aging flies before [47,48]. Further studies are needed to examine if CHD1 might actually affect the physiological age of the gut.

## Supporting Information

S1 Fig Delayed eclosion times in *Chd1*^*-/-*^ versus *Chd1*^*WT/WT*^ flies. (PDF)

S2 Fig Comparison of taxa distribution in *Chd1* wild-type and mutant guts relative to total bacterial load. (PDF)

S3 Fig Proportion of bacterial families in the individual replicates of *Chd1*^*WT/WT*^ and *Chd1*^*-/-*^ guts. (PDF)

S4 Fig Rarefaction curves for capped sequence data sets. (PDF)

S1 Table OTUs before and after filtering of OTUs with < 0.005% reads and of singletons. (XLSX)

S2 Table OTUs detected in *Chd1*^*WT/WT*^ and *Chd1*^*-/-*^ guts. (XLSX)

S3 Table Chao1 and Shannon indices of capped datasets from *Chd1* mutant and wild-type samples. (PDF)
