# Supplementary material for: Impact of the Chromatin Remodeling Factor CHD1 on Gut Microbiome Composition of Drosophila melanogaster
Source: PLoS One. 2016 Apr 19;11(4):e0153476. doi: 10.1371/journal.pone.0153476 (PMC4836739; doi:10.1371/journal.pone.0153476)
Supplement: S1 Fig — (PDF) [file pone.0153476.s001.pdf]

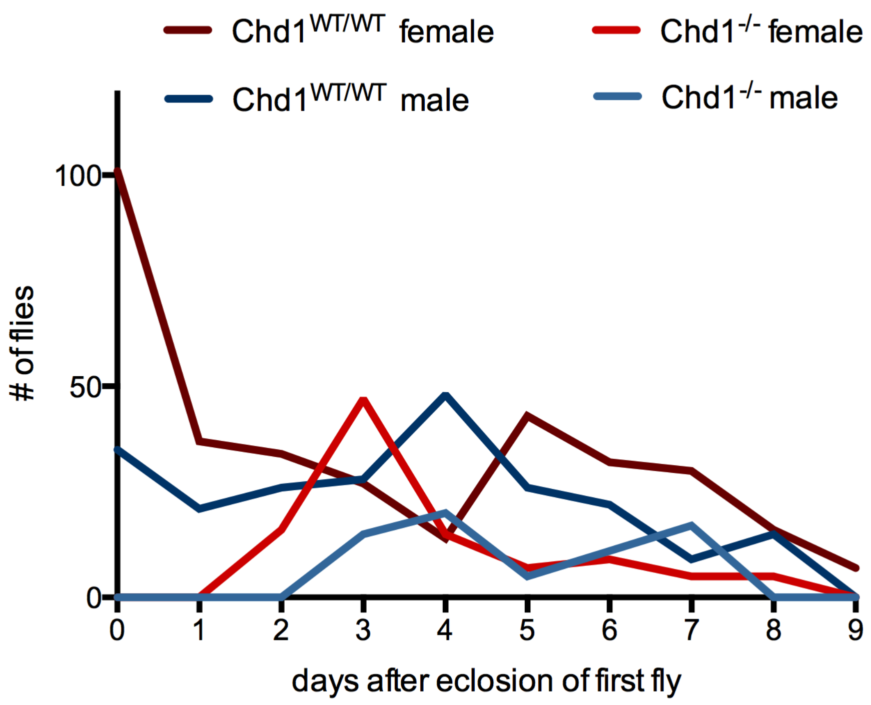

S1 Fig. Delayed eclosion times in *Chd1*$^{-/-}$ versus *Chd1*$^{WT/WT}$ flies. The following crosses were set up at the same time: *Df(2L)Chd1*^{1}*/CyO* x *Df(2L)Exel*^{7014}*/CyO* and *Df(2L)Chd1*^{1}*,P{Chd1*^{WT}*}/CyO* x *Df(2L)Exel*^{7014}*,P{Chd1*^{WT}*}*. Male and female offspring of the genotypes *Df(2L)Chd1*^{1}*/Df(2L)Exel*^{7014} ***(Chd1***$^{-/-}$***)*** and *Df(2L)Chd1*^{1}*,P{Chd1*^{WT}*}/ Df(2L)Exel*^{7014}*,P{Chd1*^{WT}*}* ***(Chd1***$^{WT/WT}$***)***, respectively, were counted at the day of their eclosion and numbers were plotted over time.
