# Supplementary material for: Impact of the Chromatin Remodeling Factor CHD1 on Gut Microbiome Composition of Drosophila melanogaster
Source: PLoS One. 2016 Apr 19;11(4):e0153476. doi: 10.1371/journal.pone.0153476 (PMC4836739; doi:10.1371/journal.pone.0153476)
Supplement: S2 Fig — (PDF) [file pone.0153476.s002.pdf]

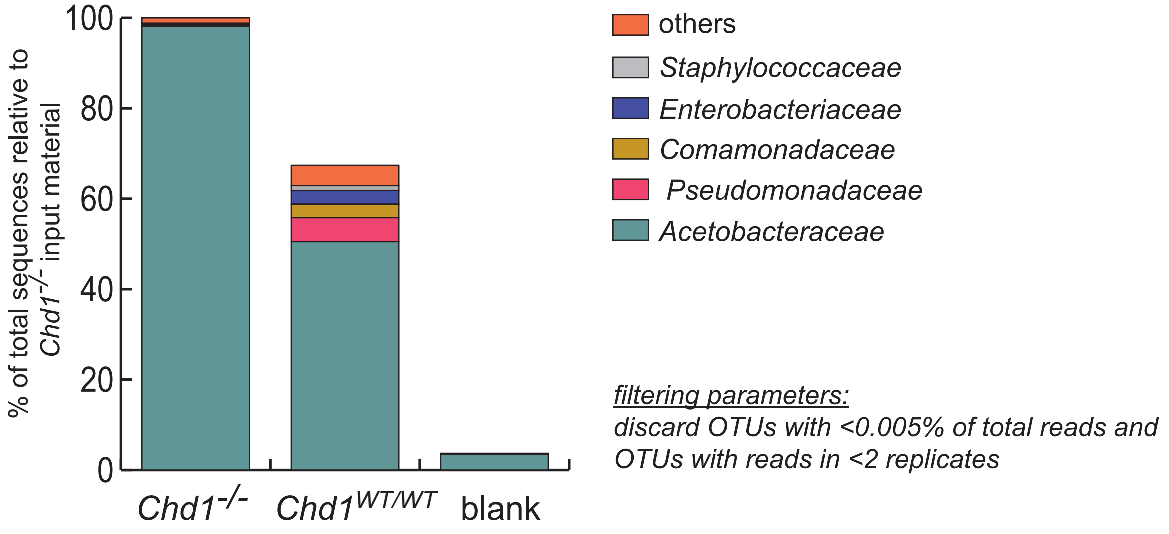

S1 Fig. Comparison of taxa distribution in *Chd1* wild-type and mutant guts relative to total bacterial load.
