# Supplementary material for: Impact of the Chromatin Remodeling Factor CHD1 on Gut Microbiome Composition of Drosophila melanogaster
Source: PLoS One. 2016 Apr 19;11(4):e0153476. doi: 10.1371/journal.pone.0153476 (PMC4836739; doi:10.1371/journal.pone.0153476)
Supplement: S3 Fig — (PDF) [file pone.0153476.s003.pdf]

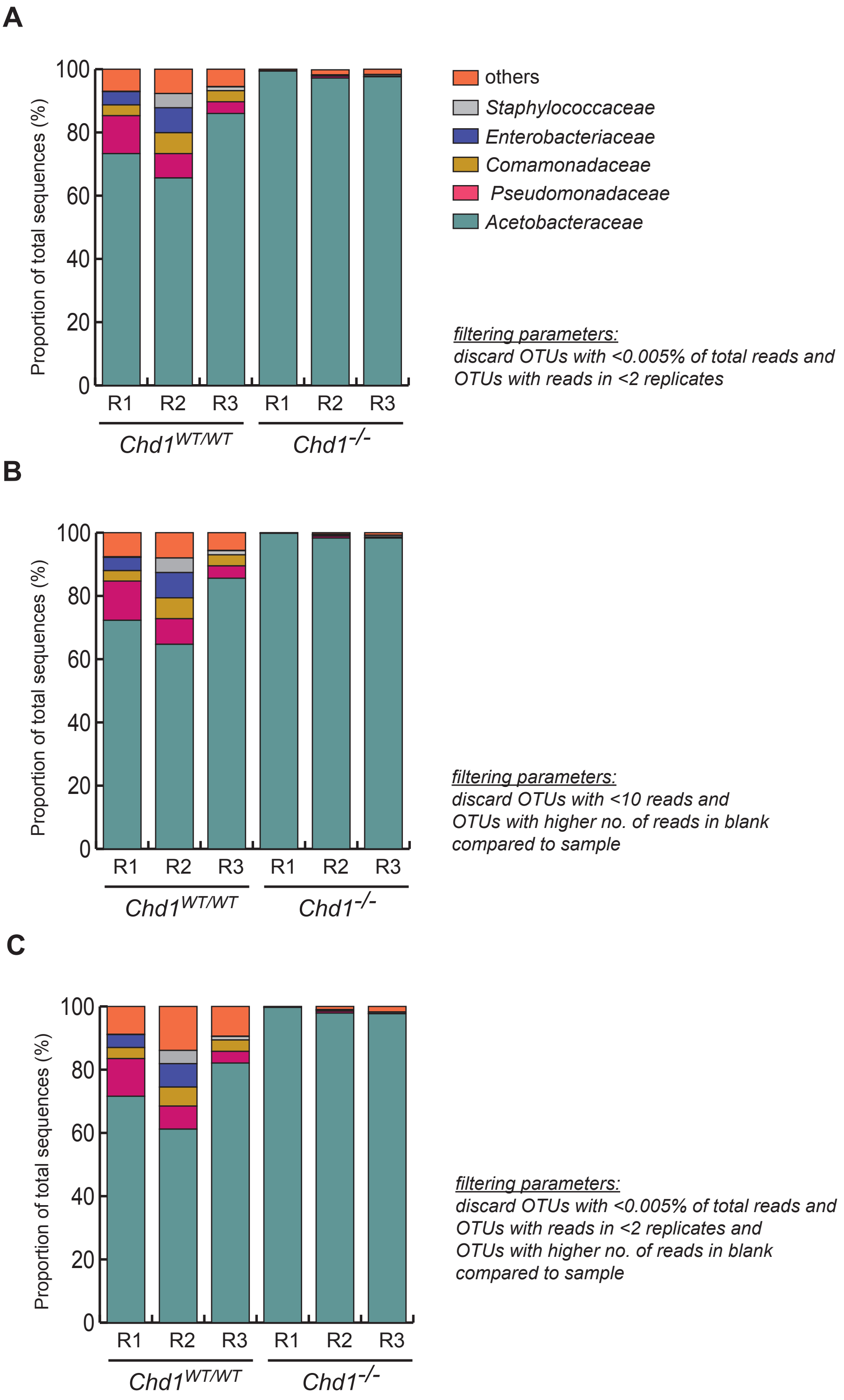

S2 Fig. Proportion of bacterial families in the individual replicates of *Chd1*$^{WT/WT}$ and *Chd1*$^{-/-}$ guts. Different filtering parameters were applied and are indicated next to the respective plots in (A)-(C).
