# Supplementary material for: Impact of the Chromatin Remodeling Factor CHD1 on Gut Microbiome Composition of Drosophila melanogaster
Source: PLoS One. 2016 Apr 19;11(4):e0153476. doi: 10.1371/journal.pone.0153476 (PMC4836739; doi:10.1371/journal.pone.0153476)
Supplement: S4 Fig — (PDF) [file pone.0153476.s004.pdf]

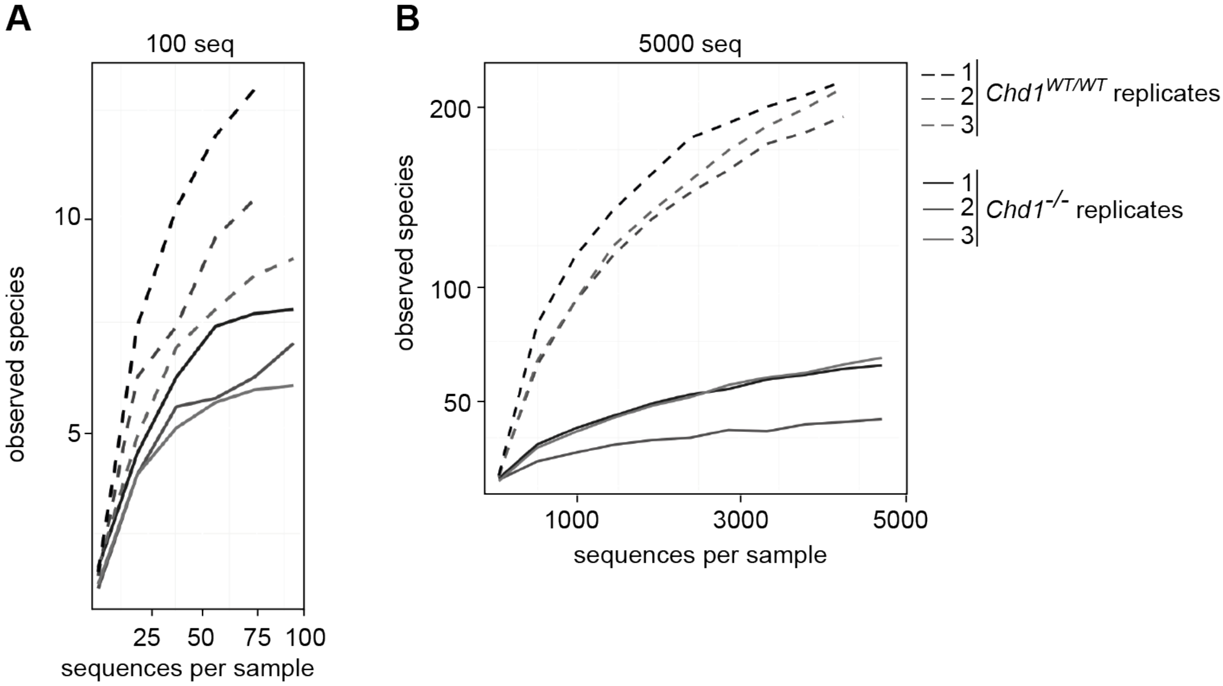

S3 Fig. **Rarefaction curves for capped sequence data sets.** (**A**) Capping the sequence number to 100 per sample indicates insufficient discriminatory power. (**B**) Capping sequence number to 5000 per sample suggests representative sampling and improves discrimination of *Chd1*$^{-/-}$ and *Chd1*$^{WT/WT}$ associated microbiota.
