# Supplementary material for: Impact of the Chromatin Remodeling Factor CHD1 on Gut Microbiome Composition of Drosophila melanogaster
Source: PLoS One. 2016 Apr 19;11(4):e0153476. doi: 10.1371/journal.pone.0153476 (PMC4836739; doi:10.1371/journal.pone.0153476)
Supplement: S3 Table — (PDF) [file pone.0153476.s007.pdf]

S3 Table. Chao1 and Shannon indices of capped datasets from *Chd1* mutant and wild-type samples. Datasets were artificially capped to 100 and 5000 reads, respectively.

		Chao1 100seq	***Shannon 100seq***	***Chao1 5000seq***	***Shannon 5000seq***
Chd1$^{WT/WT}$	rep1	12.5	1.9	244.12	3.46
	rep2	9.5	1.81	272.38	3.07
	rep3	14.5	2.72	232.72	4.56
	mean	**12.17**	**2.14**	**249.74**	**3.70**
Chd1$^{-/-}$	rep1	8.0	1.58	84.08	2.1
	rep2	7.5	1.05	44.75	1.35
	rep3	6.0	1.14	100.07	1.5
	mean	**7.17**	**1.26**	**76.3**	**1.65**
	P-value	**0.0589**	**0.0719**	**0.0016****	**0.0268***

*P-value < 0.05 is considered significant
